# Calcification in Vascular Smooth Muscle Cells Is Associated with Elevated GCLm and Impaired Contraction: Insights into Osteogenic Transdifferentiation and Therapeutic Approaches

**DOI:** 10.3390/pathophysiology32040066

**Published:** 2025-11-26

**Authors:** Luisa F. Delgadillo, Nabil A. Rashdan, Hunter Hamilton, Jack H. Pattillo, Shuai Yuan, Randa S. Eshaq, Norman R. Harris, Jonathan S. Alexander, Christopher B. Pattillo

**Affiliations:** 1 Molecular & Cellular Physiology, Louisiana State University Health Sciences Center, Shreveport, LA 71103, USA; luisa.delgadillo@lsuhs.edu (L.F.D.); nrashdan@ucdavis.edu (N.A.R.); hhamilton264462@nsula.edu (H.H.); jack.pattillo@lsuhs.edu (J.H.P.); randa.eshaq@lsuhs.edu (R.S.E.); norman.harris@lsuhs.edu (N.R.H.); jonathan.alexander@lsuhs.edu (J.S.A.); 2 Department of Pharmacology and Chemical Biology, School of Medicine, University of Pittsburgh, Pittsburgh, PA 15261, USA; yuans@pitt.edu

**Keywords:** calcification, GCLm, glutathione, antioxidant, atherosclerosis

## Abstract

Background: Vascular calcification is a strong predictor of cardiovascular morbidity and mortality. Oxidative stress plays a key role in promoting vascular calcification. Glutathione (GSH), as a major cellular antioxidant, is produced in response to oxidative stress and is regulated by the enzyme glutamate-cysteine ligase (GCL). In this study, we examined the role of the GCL modifier subunit (GCLm) in regulating vascular smooth muscle cell (VSMC) calcification. Methods: Human coronary artery VSMCs were exposed to phosphate-rich media to induce calcification. Results: Calcification led to a decrease in the GSH:GSSG ratio (reduced glutathione to oxidized glutathione), and elevated GCLm expression, coincident with mobilization of osteogenic genes and loss of contractile phenotype. KEGG pathway analysis of human unstable atherosclerotic plaques similarly showed increased GCLm expression and activation of reactive oxygen species (ROS)-related pathways. Notably, forced overexpression of GCLm in murine VSMCs (MOVAS cells) significantly accelerated calcification. These findings implicate GCLm upregulation in promoting VSMC calcification, potentially by disrupting redox homeostasis and driving phenotypic switching. Further mechanistic studies are warranted to evaluate GCLm as a potential therapeutic target in vascular calcification.

## 1. Introduction

Vascular calcification is a strong and independent predictor of morbidity and mortality for patients with conditions such as atherosclerosis, diabetes, chronic kidney disease (CKD), and aging [1]. By the age of 70, 83% of men and 71% of women exhibit arterial calcification [1]. Patients with a coronary calcium score between 101 and 300 have a 7.73-fold increased risk of a major coronary event (myocardial infarction or death due to coronary disease), and the risk for patients with a score above 300 increases to 9.67-fold [2].

Vascular calcification is an active process that shares many similarities with bone mineralization, during which vascular smooth muscle cells (VSMCs) undergo osteogenic transdifferentiation and lose their contractile properties [3]. This phenotype is characterized by decreased expression of VSMC markers such as α-smooth muscle actin, loss of mineralization inhibitors such as matrix Gla protein and sclerostin, and increased expression of osteoblast-associated markers such as osteocalcin, bone sialoprotein, and SLC20A1/Pit1 [4]. Despite these parallels, vascular calcification overlaps epidemiologically with conditions associated with impaired bone integrity, such as CKD and aging, making osteogenic pathways a limited therapeutic target [5,6]. Although many efforts have been made to elucidate the pathophysiology of vascular calcification, mechanism-based therapies remain elusive.

Oxidative stress is a common feature of the pathologies that underlie vascular calcification, including diabetes, CKD, atherosclerosis, and aging [7]. Indeed, vascular calcification has been directly associated with oxidative stress [8]. Glutathione (GSH), the cell’s primary redox buffer, plays a key role in the response to reactive oxygen species (ROS) [9], providing reducing equivalents to neutralize peroxides and hydroperoxides. GSH also performs several other critical functions, including:

- maintaining protein thiols in a reduced state to stabilize protein folding and enable cysteine modification-mediated signaling [10],
- post-translational modification of proteins by forming mixed disulfides with cysteine residues (S-glutathionylation) [11], and
- acting as a cofactor in enzymatic reactions essential for growth and cell division.

GSH is formed by glutamate-cysteine ligase (GCL), which catalyzes the formation of a peptide bond between the side chain carboxylate of glutamate and the α-amine of cysteine to form γ-glutamylcysteine, the rate-limiting step in GSH biosynthesis. The GCL holoenzyme is a heterodimer composed of a catalytic subunit (GCLc) and a modifier subunit (GCLm) [12]. GCLm binding lowers the K_m_ for glutamate and ATP while increasing the K_i_ for GSH, thereby enhancing GCLc catalytic activity [13]. GCLm expression is strongly induced by oxidative stress. Consistent with this, and given the elevated oxidative stress observed in atherosclerosis [14], we previously reported increased GCLm mRNA in unstable regions of human atherosclerotic plaques, which exhibited calcification [15]. Similarly, GCLm mRNA levels were elevated in the aortas of Klotho-deficient mice, which develop pronounced vascular calcification [16]. Unstable plaques are generally characterized by a large necrotic core and a thin fibrous cap; however, stable plaques are characterized by a thick collagen-rich fibrous cap and a smaller necrotic core [15,17,18].

While the functional role(s) of GCLm, and by extension GSH, in vascular calcification remain unclear, in this study, we evaluated the relationships between vascular calcification, GCLm expression, and human vascular tissue pathology. Using cultured cells, we investigated potential signaling pathways regulated by GCLm/GSH. As GSH has been reported to play a protective role in bone metabolism disorders, GSH regulation may also represent an attractive potential target for managing vascular calcification.

## 2. Materials and Methods

### 2.1. Cell Culture

Human Coronary Artery Smooth Muscle Cells (HCASMC), derived from the coronary artery, are primary human smooth muscle cells obtained from Promo Cell (Heidelberg, Germany; Catalog C-12511). HCASMC were cultured in Smooth Muscle Cell Growth Medium 2, which contains growth supplements specifically designed to maintain a contractile smooth muscle phenotype (smooth muscle α-actin+), calponin, and myosin heavy chain expression. These cells express extracellular matrix proteins (FN1, COL1A1, and DCN) and smooth muscle cell markers (ACTA2, MYH11, and CNN1) and were used by passage 5 to ensure maintenance of their native smooth muscle characteristics [19].

Mouse MOVAS cells were purchased from ATCC (Manassas, VA, USA; Catalog, CRL-2797) and cultured in DMEM (Gibco, MA, USA; Catalog, 12-800-017) supplemented with 10% FBS (Gibco; Catalog, 10-438-026), 1% antibiotic-antimycotic (Gibco; Catalog, 15240062), and 0.2 mg/mL G418 sulfate (Goldbio, St. Louis, MO, USA; Catalog, G-418-10). All cells were cultured in a humidified incubator at 37 °C in 5% CO_2_. This cell line is an immortalized mouse aortic vascular smooth muscle cell (VSMC) line generated via transfection with a plasmid encoding the neomycin resistance gene (neo^+^), allowing indefinite proliferation under G418 sulfate. Morphologically, MOVAS cells exhibit a spindle-shaped, smooth muscle appearance. Phenotypically, CRL-2797 expresses smooth muscle–specific markers: α-smooth muscle actin (α-SMA/ACTA2), calponin (CNN1), SM22α (TAGLN), and smooth muscle myosin heavy chain (SM-MHC/MYH11). MOVAS cells contract and proliferate in response to angiotensin II, PDGF, and TGF-β, and have been used to study vascular remodeling, inflammation, and atherosclerosis [20].

### 2.2. In Vitro Calcification

To induce calcification of smooth muscle cells (SMCs), cells were seeded at 2.5 × 10^5^ cells/well in a 12-well plate (Genesee Scientific, El Cajon, CA, USA; Catalog, 25-106) in α-MEM (Gibco; Catalog, 11-900-073) supplemented with 5% FBS (Gibco; Catalog, 10-438-026), 1% antibiotic-antimycotic (Gibco; Catalog, 15240062). On the next day (day 0), media was replaced with calcification media consisting of α-MEM (Gibco; Catalog, 11-900-073) supplemented as above with the addition of 1 M inorganic phosphate (Pi) (123.6 mM NaH_2_PO_4_ and 876.4 mM Na_2_HPO_4_, pH 7.4) to a final concentration of 3 mM Pi. For all experiments, cells were cultured for 7 days with media changes every other day; for time course experiments, calcification was initiated on day 0 (7 days), day 2 (5 days), day 4 (3 days), or on no days (control).

### 2.3. Quantification of In Vitro Calcification

To quantify calcium accumulation in cultured monolayers, cells were washed twice with 1 mL of ice-cold phosphate-buffered saline without calcium or magnesium (Fisher Scientific, Waltham, MA, USA; Catalog, AAJ67802K2). Wells were then incubated in 0.6 M HCl at room temperature for 24 h. Free calcium was quantified calorimetrically by the o-cresolphthalein method using a commercial kit (Randox Laboratories, Kearneysville, WV, USA; Catalog, CA590). The monolayers were then incubated in 0.1 M NaOH and 0.1% SDS for one hour at room temperature with vigorous shaking. The total protein concentration of monolayers was determined by the DC assay (Bio-Rad Laboratories, Hercules, CA, USA; Catalog, 5000111) and used to normalize leached calcium values.

### 2.4. Total RNA Extraction, cDNA Synthesis, and Quantitative Real-Time Polymerase Chain Reaction

Cells were lysed in Trizol reagent (Invitrogen, Carlsbad, CA, USA; Catalog, 15596018) and RNA isolated following the manufacturer’s recommendations. 1 µg of total RNA was reverse transcribed using EpiScript RNase H-Reverse Transcriptase (Lucigen, Middleton, WI, USA; Catalog, ERT12925K) and Anchored Oligo dT (IDT, Coralville, IA, USA; Catalog, 51-01-15-09) following the manufacturer’s instructions. Gene of interest cDNA was amplified in duplicate using Forget-Me-Not™ EvaGreen^®^ qPCR Master Mix (Biotium, Fremont, CA, USA). The Ct values were then normalized to the geometric mean Ct values of β-actin and Glyceraldehyde 3-phosphate dehydrogenase (GAPDH).

### 2.5. GCLm Plasmid Cloning

The coding sequences of GCLm and GFP were codon optimized by ATUM (Newark, NJ, USA) using the GeneGPS machine learning algorithm. These coding sequences were amplified by PCR, adding an EcoRI restriction site and Kozak sequence to the 5′ sequence immediately upstream of the start codon, and a NotI site to the 3′ region downstream of the stop codon. Plasmid was purified using alkaline lysis as previously published. MOVAS cells were seeded into 12-well plates at a density of 2.5 × 10^5^ cells/well. The following day, cells were transfected with 1 µg of plasmid using Lipofectamine 3000 (ThermoFisher Scientific, Waltham, MA, USA) following the manufacturer’s instructions and incubated overnight.

### 2.6. Gel Contraction Assay

Vascular smooth muscle contractility was measured in vitro using collagen gels impregnated with VSM under normal and calcifying conditions, where GSH/GCLm has been manipulated. Cell-gel mixtures using SMCs and Type I collagen, rat-tail (Enzo, Farmingdale, NY, USA; Catalog, ALX-522-435-0100) were plated on 24-well plates at a concentration of 50,000 cells/well and 2.5 mg/mL collagen in α-MEM (Gibco; Catalog, 11-900-073) for 1 h at 37 °C. After cell-gel polymerization, 1mL of media was added to each well, and the cell-gels were released from the sides of the well using a sterile 200 µL pipette tip and allowed to contract for 7 days. Images of the 24-well plate were taken each day with a Nikon camera and a plexiglass mirror platform, and contractility was analyzed as the fraction of total gel area at *t* = 0, measured using ImageJ (version 10.2) [21].

### 2.7. GSH and GSSG Measurements

Cell samples were collected in 0.5 M perchloric acid and frozen at −80 °C until processing. To process, samples were sonicated and spun at 16,000 g for 15 min. Supernatant was collected for electrochemical detection, and the pellet was resuspended in 0.1 M NaOH, 0.1% SDS, and vortexed for 15 min for protein standard analysis. Supernatant was injected into an HPLC (Shimadzu LC20 system) for column separation (C18 column), followed by electrochemical detection using a Decade Elite equipped with a FlexCell (Antec Scientific, Alphen aan den Rijn, The Netherlands).

### 2.8. KEGG Pathway Analysis

KEGG pathway analysis was performed on reanalyzed data from a previously published RNA-sequencing data set comparing stable and unstable aortic plaques using differentially expressed genes [22]. These data were previously denoted as ‘stable/unstable’ by low and high 18-F sodium fluoride tracer uptake obtained from carotid endarterectomy in symptomatic patients. Plaque instability was scored by 18F-sodium fluoride imaging of explanted plaques, and uptake of radiotracer was found to be greater in ‘unstable’ dissections compared to ‘stable’ regions [22].

### 2.9. Statistical Analysis

All data are presented as mean ± SEM. Data were analyzed by two-tailed Student’s *t*-test or one-way analysis of variance (ANOVA) followed by Tukey’s range test, as appropriate. All statistical analysis was performed using Prism 10 (GraphPad Software, Boston, MA, USA). Any *p*-values < 0.05 were considered significant, and *p*-values are represented as: * *p* <  0.05; ** *p* <  0.01; *** *p* <  0.001; **** *p* < 0.0001.

## 3. Results

### 3.1. GCLm Expression Is Upregulated During Calcification

**Figure 1:**
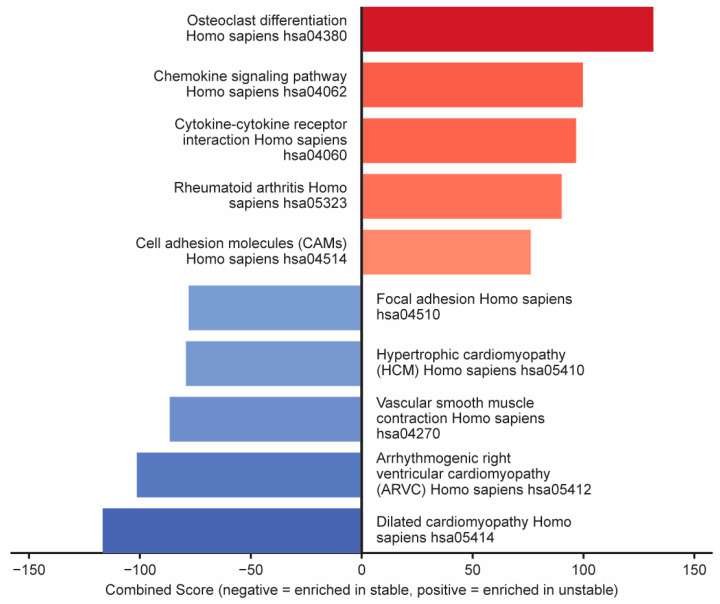
KEGG pathway analysis of differentially expressed genes in unstable compared to stable atherosclerotic plaques shows that osteoclast differentiation signaling is enriched in unstable plaques, while vascular smooth muscle contraction signaling is depressed.

KEGG pathway analysis of a previously published RNA-sequencing data set of stable vs. unstable plaques [22] revealed several upregulated pathways, which include osteoclast differentiation and cell adhesion, while downregulated pathways included vascular smooth muscle contraction and cardiomyopathy pathways (Figure 1).

**Figure 2:**
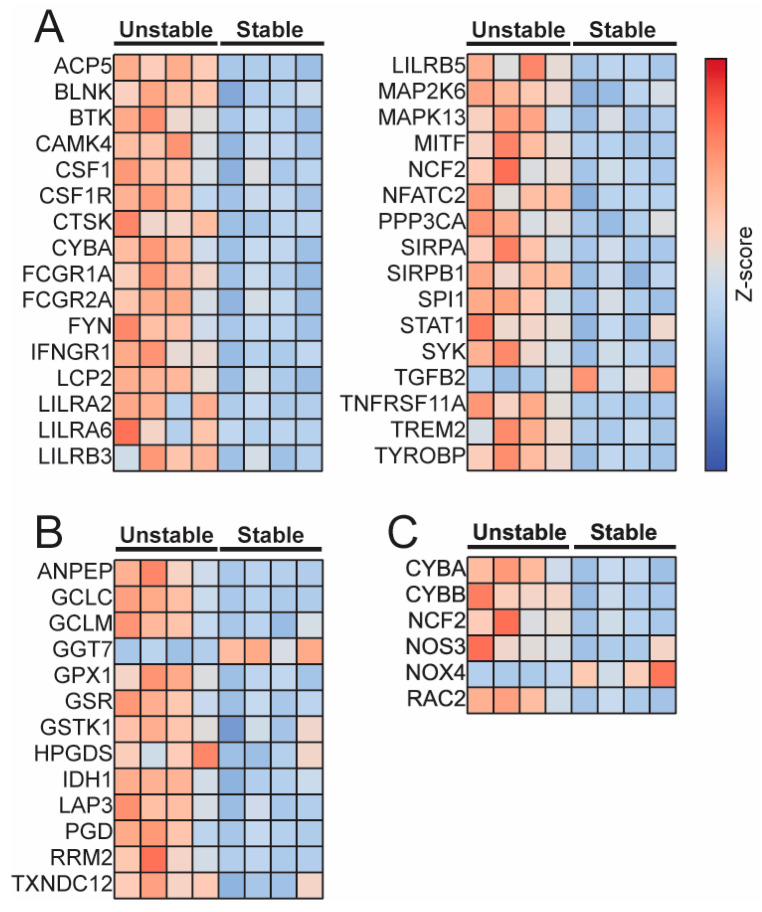
Panel (**A**)—Focusing on genes in the osteoclast differentiation pathway revealed many genes with increased expression in unstable plaques. Panel (**B**)—The glutathione metabolism pathway also contained many genes that were increased in the unstable plaques when compared to the stable plaque; among them, GCLm was increased. Panel (**C**)—Genes involved in the formation of reactive oxygen species were elevated in unstable plaques. (Gene names and functions in Supplemental Table S1).

Comparison of differentially expressed genes involved in osteoclast differentiation (Figure 2A) showed multiple genes increased in unstable plaques when compared to stable plaques. Additionally, differentially expressed genes in the glutathione metabolism pathway (hsa00480) revealed GCLm to be upregulated in unstable plaques (Figure 2B). Differentially expressed genes that contribute to reactive oxygen species (ROS) production showed an increase in genes encoding proteins important for the NADPH oxidase complex, including CYBA (cytochrome b-245 alpha chain; encodes p22^phox^), CYBB (cytochrome b-245 beta chain; encodes p91^phox^), and NCF2 (neutrophil cytosolic factor 2; encodes p67^phox^) (Figure 2C).

**Figure 3:**
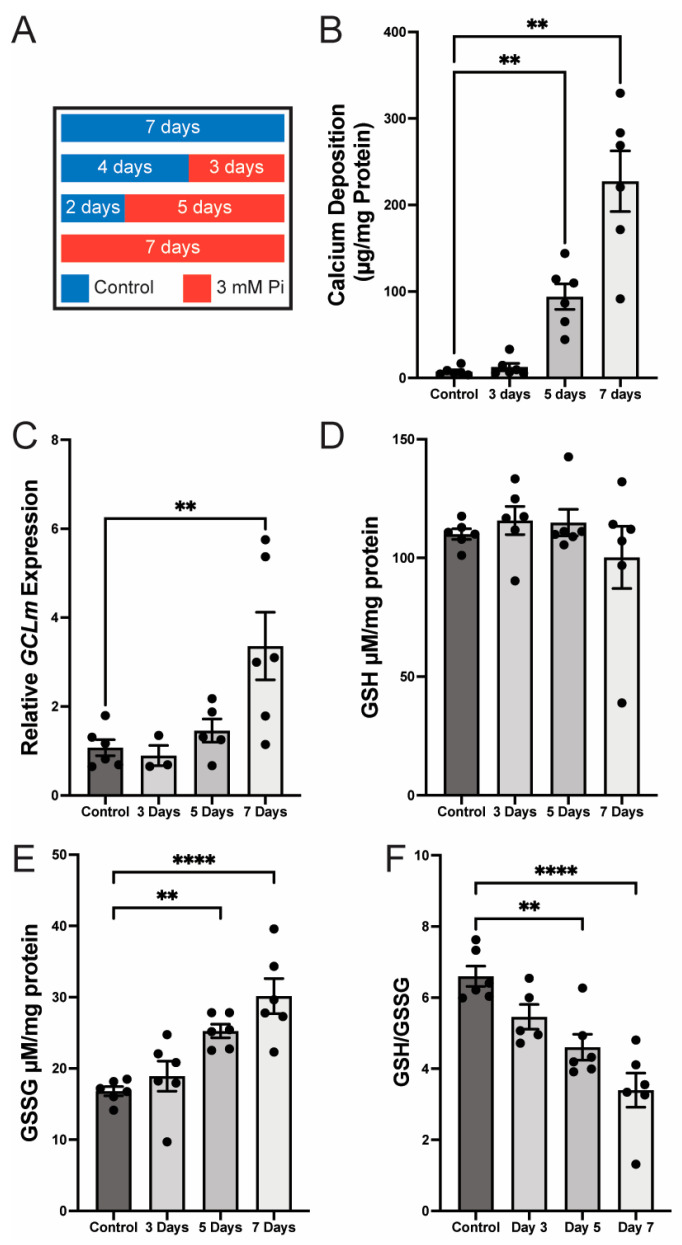
Panel (**A**)—Scheme demonstrating the smooth muscle cell treatment to drive calcification. Panel (**B**)—Calcium deposition in smooth muscle cells increases over the duration of treatments. Panel (**C**)—*GCLm* expression in smooth muscle cells increases during calcification. Panel (**D**)—Glutathione (GSH) concentrations remain the same during calcification. Panel (**E**)—Glutathione disulfide (GSSG) levels increase during calcification. Panel (**F**)—Glutathione antioxidant potential (GSH:GSSG) decreases during calcification. (** *p* < 0.01, **** *p* < 0.0001, *n* = 5–6 [panel (**C**) day 3 has *n* = 3]).

Smooth muscle calcification (based on the scheme shown in Figure 3A) was modeled using HCASMC cultured in 3 mM Pi media for up to 7 days. Three mM Pi significantly increased calcium deposition at days 5 and 7 (17.09 and 41.36-fold, respectively, *p* < 0.01) (Figure 3B). The increase in calcium deposition was accompanied by a significant relative increase in GCLm mRNA (3.36-fold of control, *p* < 0.01) (Figure 3C). Despite the increase in GCLm mRNA at day 7, GCLc (the catalytic subunit of GCL) was significantly elevated at day 5 but not at day 7. As GCLm was the focus of this manuscript, we did not pursue GCLc (Supplemental Figure S1). There was no change in glutathione (GSH) levels (Figure 3D); however, there was a significant increase in glutathione disulfide (GSSG) on days 5 and 7 (Figure 3E), resulting in a significant decrease in the ratio of GSH:GSSG on days 5 and 7 (Figure 3F). Because calcification was associated with a marked upregulation of GCLm mRNA (3.36-fold increase), GCLm expression is highly responsive to VSM calcifying conditions. Notably, while total GSH levels remained unchanged, there was a significant rise in GSSG, the oxidized form of glutathione, on days 5 and 7. As a result, the GSH:GSSG ratio—a marker of cellular redox balance—declined significantly, indicating increased oxidative stress during the calcification process. These findings suggest that phosphate-induced VSMC calcification is accompanied by redox imbalance and selective upregulation of GCLm, implicating oxidative stress and glutathione pathway dysregulation as potential contributors to the calcific phenotype.

**Figure 4:**
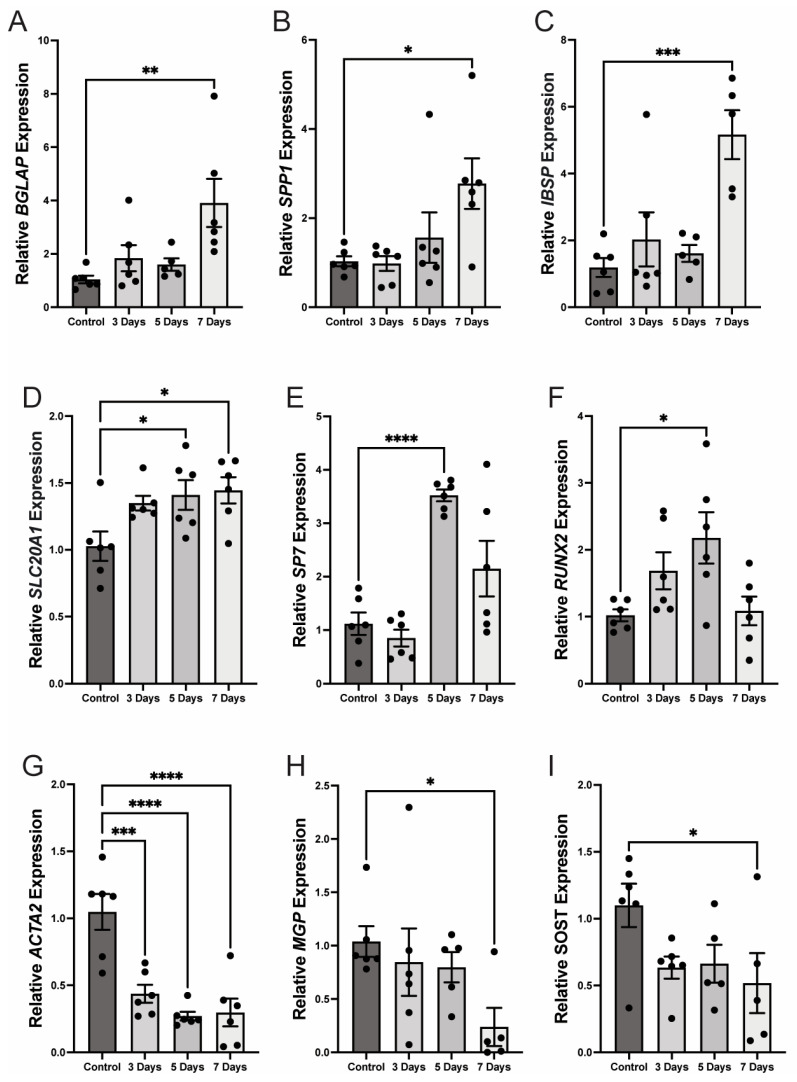
Panels (**A**–**F**)—Osteogenic marker expression increases in smooth muscle cells undergoing calcification. Panel (**G**)—A classic marker of smooth muscle cells is decreased in smooth muscle cells as they calcify. Panels (**H**,**I**)—Expression of two mineralization inhibitors is depressed during calcification. (* *p* < 0.05, ** *p* < 0.01, *** *p* < 0.001, **** *p* < 0.0001, *n* = 5–6).

As previously reported, culturing HCASMCs in 3 mM phosphate (Pi) calcifying medium induced osteogenic transdifferentiation, as was shown by significant upregulation of multiple osteogenic markers: these included *BGLAP* (osteocalcin; 3.91-fold increase, *p* < 0.01), a hormone secreted primarily by osteoblasts; *SPP1* (osteopontin; 2.78-fold, *p* < 0.05), a glycoprotein involved in bone remodeling; *IBSP* (bone sialoprotein; 5.16-fold, *p* < 0.001), a bone matrix-associated glycoprotein; *SLC20A1* (1.45-fold, *p* < 0.05), sodium-dependent phosphate transporter which drives cellular phosphate uptake); SP7 (osterix; 3.52-fold, *p* < 0.001), a central transcription factor which can drive osteoblast differentiation; and *RUNX2* (2.18-fold, *p* < 0.05), the ‘master’ transcriptional regulator of osteogenesis (Figure 4A–F).

Conversely, expression of *ACTA2* (α-smooth muscle actin; 0.27-fold, *p* < 0.001), a key marker of the smooth muscle contractile phenotype, was significantly reduced under these conditions (Figure 4G). Further, two other important inhibitors of mineralization—*MGP* (matrix Gla protein; 0.24-fold, *p* < 0.05) and *SOST* (sclerostin; 0.52-fold, *p* < 0.05)—are also markedly downregulated during experimental calcification (Figure 4H,I).

Taken together, our findings show that exposure to elevated phosphate drives HCASMCs toward an osteogenic phenotype while suppressing expression of contractile and anti-calcification genes. Importantly, these changes occur in parallel with increased *GCLm* expression, potentially linking redox regulation to the molecular reprogramming that underlies smooth muscle cell calcification.

**Figure 5:**
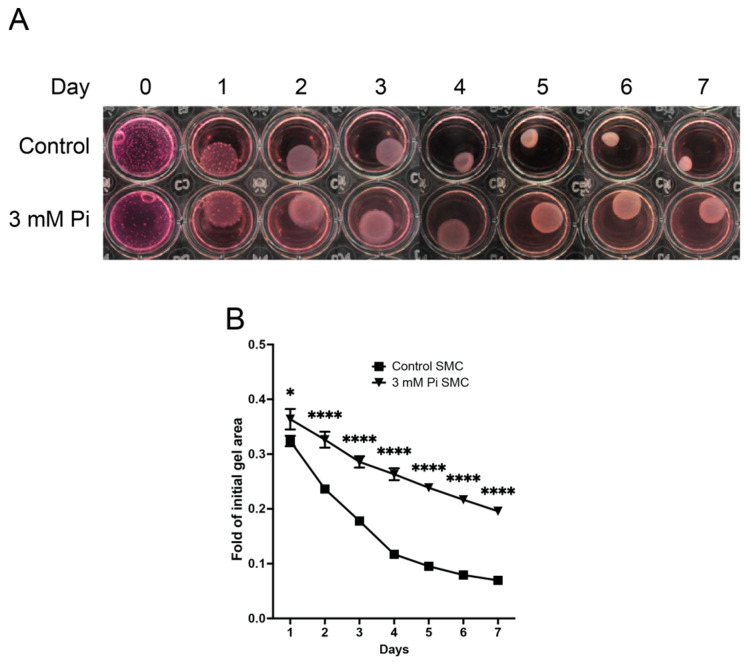
Panel (**A**)—Collagen gel with smooth muscle cells over 7 days. Panel (**B**)—Smooth muscle contraction is depressed as cells calcify. (* *p* < 0.05, **** *p* < 0.0001, *n* = 6).

To functionally evaluate this expression profile, collagen gel contraction assays were performed on HCASMCs. Collagen gel contraction was significantly less in cell-gels cultured in 3 mM Pi compared to cell-gels cultured in control media across all times measured, day 1 (*p* < 0.05), day 2–7 (*p* < 0.001) (Figure 5A,B).

### 3.2. TGF-β Expression Increases in Calcifying HCASMC

**Figure 6:**
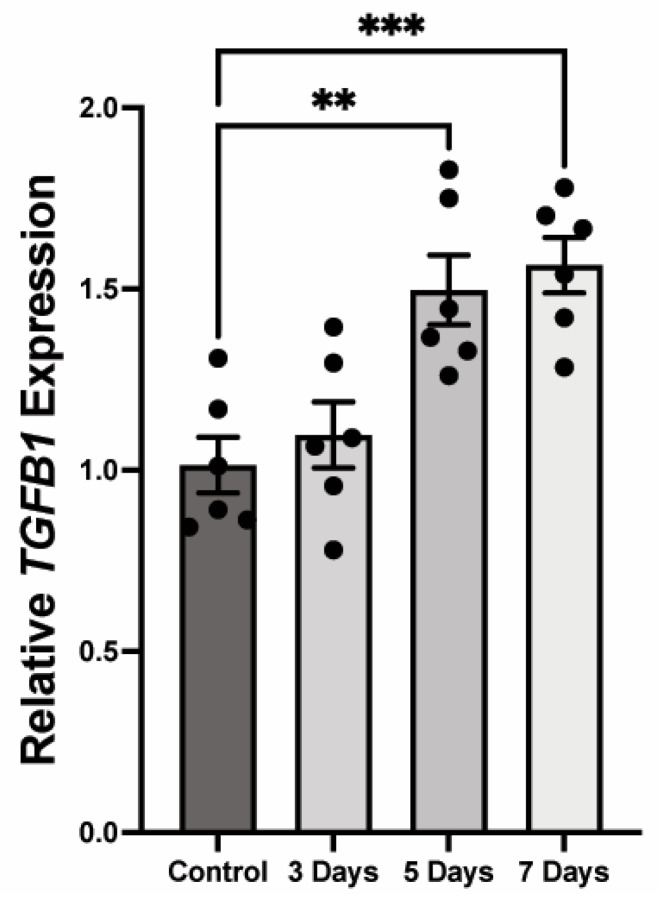
*TGFβ* expression increases as smooth muscle cells calcify. (** *p* < 0.01, *** *p* < 0.001, *n* = 6).

A key upstream regulator of collagen gel contraction is transforming growth factor beta (TGFβ) signaling [23]. To determine the effect of these changes on HCASMC calcification, we observed that 3 mM Pi induced a significant increase in relative *TGFβ1* at 5 (1.5-fold, *p* < 0.01) and 7 (1.57-fold, *p* < 0.001) days compared to control (Figure 6).

### 3.3. Elevated GCLm Increases Calcification of MOVAS

**Figure 7:**
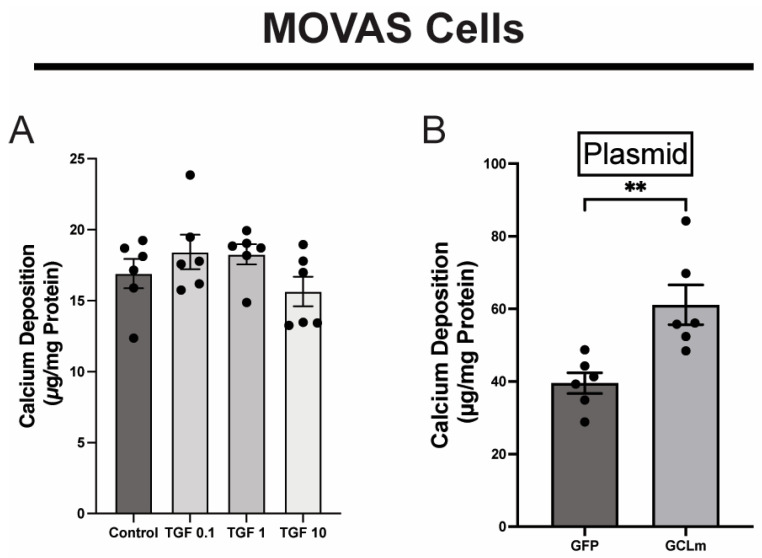
Panel (**A**)—Addition of exogenous TGFβ did not increase calcification (7 days). Panel (**B**)—Overexpression of GCLm resulted in increased calcification. (day 7; ** *p* < 0.01, *n* = 6).

To assess the relative contributions of TGFβ and GCLm to calcification, we used MOVAS cells, a mouse aortic smooth muscle cell. We first treated calcifying MOVAS cells with increasing concentrations of TGFβ and saw no increase in calcification over vehicle treatment (Figure 7A). Next, we expressed either GFP or *GCLm* in calcifying MOVAS cells and observed a significant increase in calcium deposition in response to GCLm (Figure 7B). Taken together, these data suggest that GCLm contributes to vascular smooth muscle calcification more than TGFβ.

## 4. Discussion

GCLm is a crucial regulator of GSH synthesis, which governs diverse cellular defensive and phenotypic regulatory mechanisms. Our current study addresses the hypothesis that GCLm may influence vascular calcification through mechanisms that include loss of SMC differentiation. To investigate this, the expression of genes related to the osteogenic differentiation pathway, glutathione metabolism, as well as ROS-producing genes, was compared in stable vs. unstable atherosclerotic plaques. Interestingly, our analysis showed an increased expression of several calcification-associated genes, along with elevated expression of glutathione metabolism genes, particularly GCLm, in unstable plaques. This increase in glutathione metabolism genes may reflect a cellular adaptation to an abundance of ROS. These results parallel those reported by Zhang et al., who demonstrated that VSMCs undergo phenotypic switching from a contractile phenotype to a synthetic phenotype through oxidative stress-induced PARP1 signaling in a MOVAS cell model of calcification [24].

In an effort to evaluate whether and to what extent ROS genes might also be elevated during calcification, we considered the expression of genes commonly induced by exposure to elevated ROS. We observed that genes encoding the NADPH oxidase complex increased, as well as endothelial nitric oxide synthase, which was elevated. While NADPH oxidase and NO synthase (NOS) are both capable of producing superoxide [25] NOS is more typically associated with nitric oxide production, which, with superoxide, can yield peroxynitrite [26]. Interestingly, NADPH oxidase (NOX4) has been shown to be required for maintaining the normal smooth vascular muscle cell phenotype [27].

When we studied unstable plaques, we found a decrease in NOX4, which paralleled elevations in osteogenic genes. Future work in this field should consider how ROS-producing genes influence ROS generation and modulate signaling through ROS-dependent and independent pathways.

In addition to evaluating osteogenic pathway activity, glutathione-related genes, and ROS-producing genes, we established an in vitro model of calcification in human smooth muscle cells (SMCs). These data suggest that vascular calcification is associated with increased GCLm in arterial smooth muscle; however, the nature of this interaction remains unclear. Notably, GCLm mRNA is not significantly elevated at day 5 but shows significant upregulation by day 7 (Figure 3C) in parallel with increased GSSG and a reduced GSH:GSSG ratio (Figure 3F). While total GSH levels remained unchanged, the rise in GSSG at later stages of calcification is consistent with enhanced conversion of GSH to GSSG, reflecting ROS scavenging. Together, these findings support a model in which GCLm induction represents an adaptive/permissive response to ongoing oxidative stress, where GCLm upregulation contributes to the progression, rather than initiation of arterial calcification.

Similarly to findings in the unstable and stable plaque data, calcifying SMCs showed increases in osteogenic marker expression. There was also a decrease in SMC marker expression, which was also associated with decreased SMC contraction in a collagen gel contraction assay [28,29]. This approach provided a quantitative evaluation of cell differentiation as a function of calcification, GCLm, and GSH status. Consistent with osteogenic transition, calcifying SMCs exhibited significantly reduced contractile capacity, supporting the observed phenotypic shift.

Given the well-established role of TGF-β signaling in regulating SMC differentiation, we also found that TGF-β was upregulated during calcification. Surprisingly, the exogenous addition of TGF-β did not result in an increase in calcification. This could suggest a limit on the effect of TGF-β or possibly a saturation of the system; further studies are required to determine the reason that there was no increase in calcification. Conversely, an increase in GCLm resulted in an increase in calcification, suggesting that GCLm plays a role in calcification that has not been previously reported. A publication by Callegari et al. demonstrated that overexpression of macrophage GCLc resulted in an increase in total glutathione content (GSH + GSSG) and a decrease in atherosclerotic calcification [30], but to date, no one has shown a link between GCLm and calcification.

**Figure 8:**
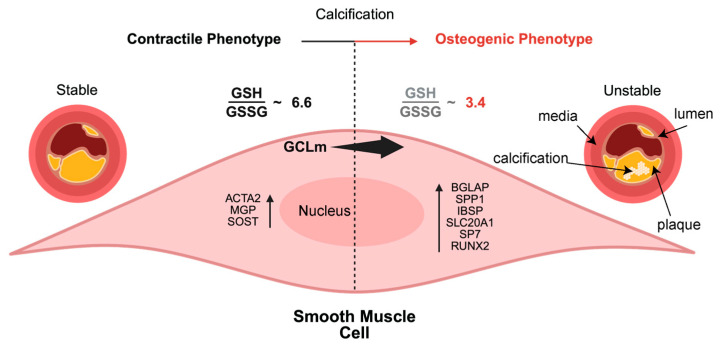
Schematic representation of our findings in this manuscript illustrating the change in smooth muscle cells from a contractile phenotype to a more osteogenic profile under calcifying conditions. Aortic cross sections are shown with plaques in yellow, calcification in white, the media is the location of the smooth muscle cells under normal conditions, and the lumen is the interior of the vessel.

At first, this finding may appear to conflict with our findings; however, it is important to remember that in our present study, we are increasing the modifier subunit of the GCL enzyme (GCLm), and the above-referenced manuscript was overexpressing the catalytic subunit GCLc. GCLm is known to enhance the catalytic efficiency of GCLc; however, isolated overexpression without matched increases in GCLc or substrate availability may not improve antioxidant capacity [31] and may even disrupt holoenzyme regulation. Our model is consistent with excess modifier subunit (GCLm) disrupting holoenzyme assembly or feedback inhibition, leading to deficient GSH synthesis, which intensifies oxidative stress. Additionally, our previous work demonstrated that aortic and venous endothelial cells exhibit fundamentally different redox handling capacities reflected through GSH and GSSG levels [32]. This highlights the importance of cellular context in oxidative stress responses and suggests that macrophages and smooth muscle cells may differ in their responses to alterations in local ROS levels. Consequently, cell–type–specific factors must be considered when interpreting the role of GCLm and redox signaling in maintaining vascular calcification. Our findings for this manuscript are summarized in Figure 8.

To the best of our knowledge, this is the first study demonstrating increases in GCLm expression in the context of VSMC calcification, highlighting new avenues of potential therapeutic strategies for the inhibition of vascular calcification. However, several technical and sample limitations exist that reveal new opportunities to extend our findings. Future studies on GCLm overexpression, knockdown, and in vivo genetic deletion models in human cell models, which are beyond the technical scope of this report, could address the role of GCLm in a more rigorous manner and define the relationships between GCLm and calcification. A genetic model of smooth muscle cell-specific deletion of GCLm would also provide us with another cell model to overexpress GCLm; in this project, we used MOVAS cells for the ease of genetic manipulation and passaging; however, they may not fully recapitulate the phenotype of primary human SMCs, despite their previous use in calcification assays [33,34]. A limitation of this study is the lack of direct quantification of GCLm protein levels and redox status in the overexpression model. Future experiments will address this gap by assessing enzyme activity, GSH flux, and ROS levels in cells with modulated GCLm expression. In vivo studies, smooth muscle cells could help account for any inconsistencies or loss of phenotype encountered in cell culture. In this study, we have shown that GCLm expression was associated with calcification and contractility; further outlined studies (described above) may provide additional and key mechanistic links between expression and calcifying markers. While our MOVAS overexpression model confirmed the role of GCLm in calcification, we did not assess GSH/GSSG levels in this system. Finally, pharmacological inhibition of GCL would represent an orthogonal approach to test whether suppression of GCL activity attenuates calcification. These experiments are of high interest and will be addressed in future studies. Nonetheless, the present work demonstrates, through convergent evidence in human Pi-treated HCAMSCs and a genetic gain-of-function model, that upregulation of GCLm is consistently associated with increased glutathione synthesis and calcification. We therefore conclude that GCLm helps to modulate arterial smooth muscle calcification. Future studies relating arterial smooth muscle redox, GCLc, and GCLm function may provide useful insight into how calcification is induced and maintained, revealing novel therapeutic targets in cardiovascular disease.

## Data Availability

The data set is available on request from the authors.

## Supplementary Materials

The following supporting information can be downloaded at: https://www.mdpi.com/article/10.3390/pathophysiology32040066/s1, Supplemental Table S1: Gene names and functions from Figure 2. Supplemental Figure S1: *GCLc* expression in smooth muscle cells is increased on day 5.
