# Supplementary figures and images for: Calcification in Vascular Smooth Muscle Cells Is Associated with Elevated GCLm and Impaired Contraction: Insights into Osteogenic Transdifferentiation and Therapeutic Approaches
Source: Pathophysiology. 2025 Nov 26;32(4):66. doi: 10.3390/pathophysiology32040066 (PMC12736360; doi:10.3390/pathophysiology32040066)

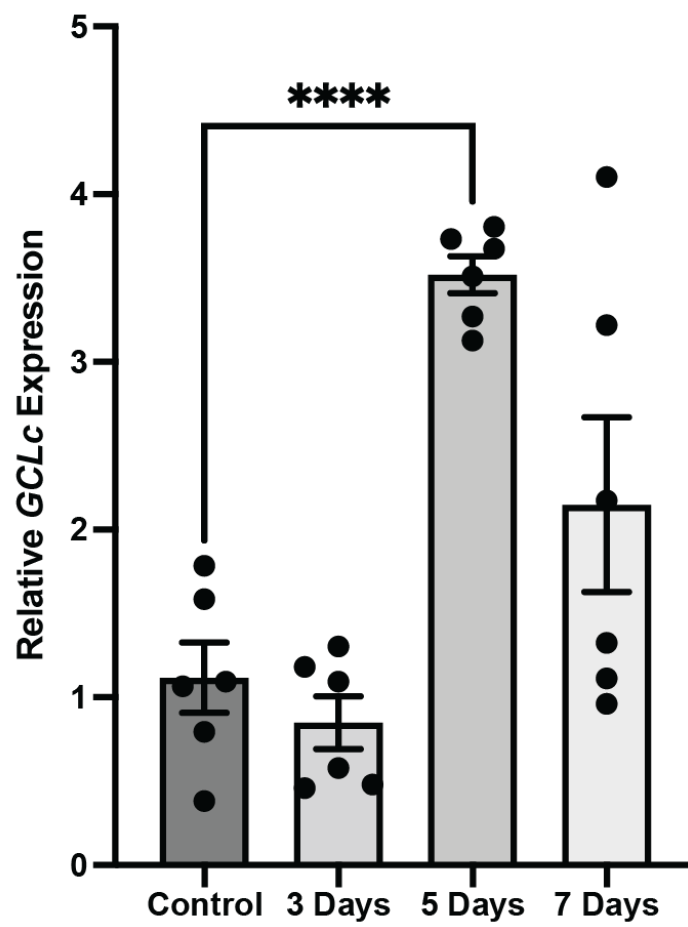

**Figure S1.** : *GCLc* expression in smooth muscle cells is increased on day 5.

Supplement: Supplementary file 1 [file pathophysiology-32-00066-s001.zip › Supplementary Figure.pdf]
